# The complete mitochondrial genome of the green microalgae *Dunaliella salina* strain SQ

**DOI:** 10.1080/23802359.2017.1331331

**Published:** 2017-05-27

**Authors:** Dante Magdaleno, Haydee Lopez, Jose Luis Stephano Hornedo

**Affiliations:** a Facultad de Ciencias Marinas, Universidad Autonoma de Baja California, Ensenada, BC, Mexico;; b Facultad de Ciencias, Universidad Autonoma de Baja California, Ensenada, BC, Mexico

**Keywords:** *Dunaliella salina*, mitochondrial genome, microalgae

## Abstract

The complete mitochondrial genome of the microalgae *Dunaliella salina* strain SQ (GenBank accession number: KX641170) was *de novo* assembled and annotated using Illumina MiSeq sequencing data. The mitochondrial genome is 41,904 bp long with 31.85% GC content and contains 7 protein-coding genes, 16 introns, 3 ribosomal RNA genes and 3 transfer RNA genes. To date, only two complete mitochondrial genomes of *Dunaliella salina* strains have been reported, and this genome provides knowledge to the study of genetic variations and evolution of mitochondrial genomes of *Dunaliella salina* strains.

Green microalgae biomass has been used for decades; in recent years, secondary metabolites extracted from green microalgae have gained interest for their commercial and biotechnological potential. The unicellular microalgae *Dunaliella salina* lives in hypersaline environments and under stress conditions can produce about 10% β-carotene of biomass dry weight (Lamers et al. 2008). β-carotene is an important pro-vitamin A, and vitamin A is essential to human body for maintaining normal vision, tissue differentiation and immune competence (Weber & Grune 2012; Sommer & Vyas 2012). To date, only two complete mitochondrial genomes of *D. salina* strains have been reported: *D. salina* CCAP 19/18 (Smith et al. 2010) and *D. salina* CONC-001 (Del Vasto et al. 2015); these genomes present several differences in genome size and GC content between strains. Here we report the complete mitochondrial genome of *D. salina* SQ to provide information about the genome architecture between *D. salina* strains.

*Dunaliella salina* was collected from a hypersaline lagoon in San Quintin, Baja California, México (30° 32’ 13.91" N, 116° 2’ 1.22" W). A single cell was isolated and cultured in modified liquid medium (Feng et al. 2009) with 250 mM NaCl. Mitochondrial DNA was extracted with AxyPrep Multisource Genomic DNA Miniprep Kit (Axygen Biosciences, Union City, CA), and mtDNA was sequenced using Illumina MiSeq (UC, San Diego, CA) obtaining 300-bp paired-end reads. The genome was *de novo* assembled using A5-miseq pipeline (Coil et al. 2014) and Ray v2.3.2 (Boisvert et al. 2010). Annotation of the mitochondrial genome was performed with RNAweseal (Lang et al. 2007), MFannot (Beck & Lang 2010) and manual curated, and results were validated using BLAST searches. The annotated genome has been deposited in the GenBank database under accession number KX641170. Whole mitochondrial genome sequence of *D. salina* has a circular structure of 41,904 bp, containing 7 protein-coding genes, 16 introns, 3 ribosomal RNA genes and 3 transfer RNA genes. The contents of A, G, T and C are 32.6%, 16.7%, 35.5% and 15.2%, respectively, with 31.85% of GC content. All protein-coding genes started with ATG and ended by TAA as a stop codon, except *nad6* which ended by TGA. The *nad1* gene contains 1 intron and 1 intronic open reading frame GIY-YIG homing endonuclease, *nad4* 1 intron, *cob* gene 5 introns and 1 intronic open reading frame LAGLIDADG homing endonuclease, *cox1* gene 7 introns and 2 intronic open reading frames LAGLIDADG homing endonuclease, *nad5* gene 2 introns and 1 open reading frame GIY-YIG homing endonuclease. For the phylogenetic analysis, 10 mitochondrial genome sequences from other microalgae were selected and downloaded from NCBI database and a neighbour-joining tree with 500 bootstraps was constructed using MEGA7 (Kumar et al. 2016) (Figure 1).

**Figure 1.**
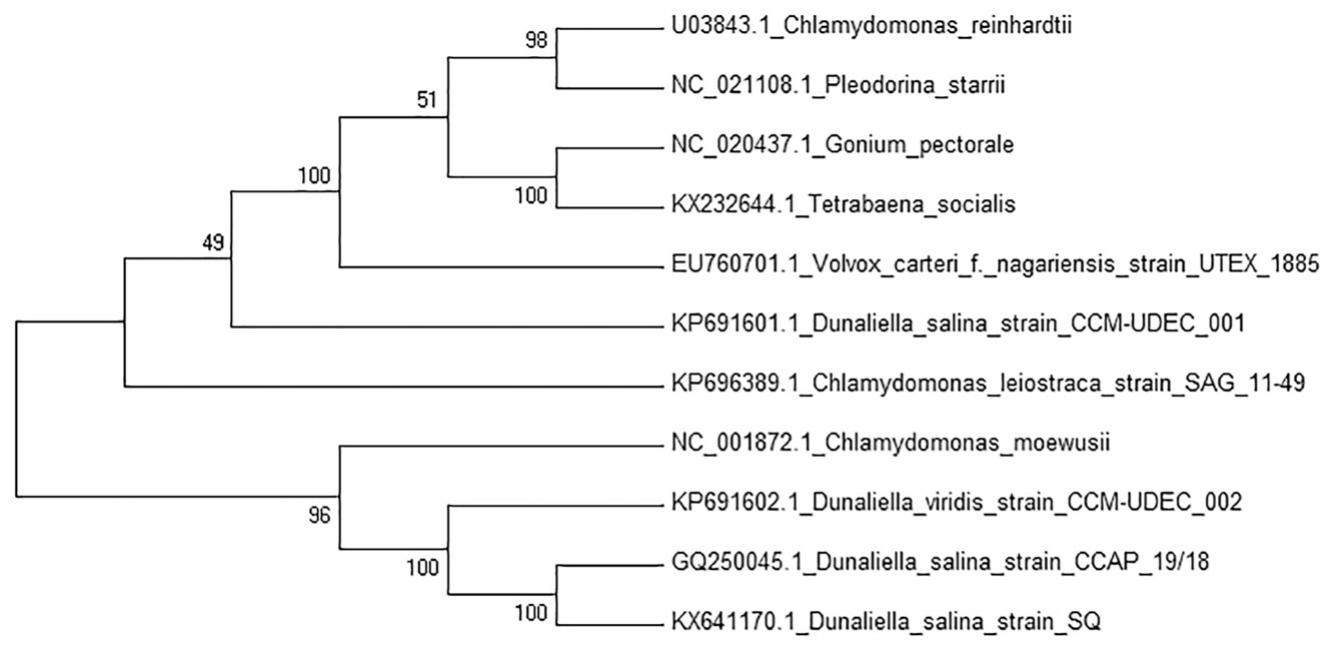
Phylogenetic tree of *D. salina* SQ and 10 microalgae mitochondrial genomes including two other strains of *D. salina,* based on the NJ method.
